# Oxidative stress-mediated responses in endometrial cancer cells: contrasting effects of doxorubicin and menadione

**DOI:** 10.3389/fphys.2026.1733194

**Published:** 2026-02-02

**Authors:** Joanna Kozak, Sandra Tkaczyk–Beraś, Krzysztof Jędraszek

**Affiliations:** Department of Basic Medical Sciences, Institute of Medical Sciences, Faculty of Medicine, The John Paul II Catholic University of Lublin, Lublin, Poland

**Keywords:** doxorubicin, endometrial cancer, menadione, oxidative stress, SESN2, SESN3, SOD1

## Abstract

**Background:**

Oxidative stress plays a crucial role in the development and treatment response of endometrial cancer, yet the antioxidant defense mechanisms in different tumor subtypes remain unclear.

**Methods:**

We investigated the cellular response to oxidative (menadione) and genotoxic (doxorubicin) stress in two TP53-mutated endometrial cancer cell lines, AN3CA and KLE. Cell viability, reactive oxygen species (ROS) levels, and the expression of antioxidant-related genes (SESN2, SESN3, SOD1) were assessed using qPCR and In-Cell Western assays.

**Results:**

AN3CA cells showed greater sensitivity to doxorubicin, marked by increased ROS and reduced viability, while KLE cells were more susceptible to menadione-induced toxicity. Protein expression analysis revealed a biphasic response: low doses of doxorubicin transiently increased SESN and SOD1 expression, whereas higher doses suppressed them. Gene expression at the mRNA level did not always correlate with protein levels, suggesting possible post-transcriptional regulation.

**Conclusion:**

Our findings demonstrate cell line - specific redox responses and identify SESN2, SESN3, and SOD1 as key players in the antioxidant defense network. These genes may serve as potential therapeutic targets in aggressive, hormone-independent endometrial cancers.

## Introduction

Endometrial cancer (EC) is one of the most common gynecological malignancies. Traditionally, EC has been described using a dualistic model that distinguishes type I tumors (predominantly endometrioid, estrogen-dependent, usually well differentiated and associated with a more favorable prognosis) from type II tumors (typically non-endometrioid, most notably serous, hormone-independent, high grade, and clinically aggressive) (Bokhman, 1983). Type II EC, in particular, represents a clinically aggressive subtype associated with poor prognosis and frequent TP53 mutations (Theisen et al., 2014; Kozak et al., 2018). However, molecular studies have demonstrated that this binary classification does not fully capture the biological heterogeneity of EC. In 2013, The Cancer Genome Atlas (TCGA) proposed a genomics-based classification comprising four major molecular subgroups: (i) POLE-ultramutated tumors, characterized by pathogenic mutations in the exonuclease domain of POLE and an excellent prognosis; (ii) MSI-high/mismatch repair–deficient (MMRd) tumors, defined by defects in DNA mismatch repair, hypermutation, and an intermediate prognosis; (iii) copy-number high/TP53-mutant (p53-abnormal) tumors, representing the most aggressive biology; and (iv) copy-number low/NSMP tumors, typically associated with more favorable outcomes and often corresponding to classic type I endometrioid cancers (Levine et al., 2013; WHO Classification of Tumours Editorial Board, 2020; Santoro et al., 2024). Among available preclinical models, the KLE and AN3CA cell lines are widely used to study clinically aggressive EC due to their hormone receptor negativity (ER–/PR–), high-grade histology, and assignment to the p53-abnormal (p53abn) and MMRd TCGA subgroups, respectively (Skok et al., 2020; Li et al., 2025). Their lack of hormone receptor expression, altered PTEN function, and disruptions in PI3K signaling make them particularly valuable for investigating receptor-independent therapeutic strategies (Korch et al., 2012; Yang et al., 2014).

DOX is a topoisomerase II inhibitor belonging to the anthracycline class and is widely employed in the treatment of various malignancies, including endometrial and ovarian cancers. Its anticancer activity stems from multiple interrelated mechanisms. First, DOX intercalates into DNA and inhibits topoisomerase II, leading to DNA double-strand breaks, genomic instability, and cell death (Fabi et al., 2021; Yang et al., 2021; Kciuk et al., 2023). Second, DOX promotes oxidative stress through redox cycling of its quinone moiety. Mechanistically, DOX may undergo one-electron reduction to a semiquinone free radical catalyzed by flavin-containing reductases and related oxidoreductases; under aerobic conditions, the semiquinone reacts rapidly with molecular oxygen, generating superoxide and sustaining redox cycling. In particular, DOX can be reduced at the quinone residue by cytochrome P450 reductase in the presence of NADPH, yielding the semiquinone radical and superoxide generation; superoxide has also been linked to oxidative activation of DOX, including one-electron oxidation involving the para-hydroxy residue (Navarro et al., 2006). Moreover, several ROS-generating routes have been proposed in mitochondria: DOX redox cycling may be coupled to the mitochondrial respiratory chain, and both enzymatic pathways (including cytochrome P450–related activities, NAD(P)H dehydrogenases, and endothelial nitric oxide synthase) and non-enzymatic, iron-dependent reactions have been implicated in ROS formation (Kim et al., 2006). ROS accumulation contributes not only to apoptosis but also to senescence and autophagy, and in some contexts the excessive accumulation of ROS during chemotherapy may sensitize cancer cells to interventions that further augment ROS production (Kozak et al., 2020; Kciuk et al., 2023). Despite its potent therapeutic effect, the clinical use of DOX is limited by dose-dependent cardiotoxicity, for which oxidative injury is a leading proposed mechanism, particularly involving mitochondrial ROS generation and redox-active iron (Zhou et al., 2001; Kim et al., 2006; Zhang et al., 2012). Efforts to mitigate this toxicity have included development of liposomal formulations, prodrug conjugates, and antioxidant co-therapies, though with mixed clinical success (Kciuk et al., 2023).

Menadione, also known as vitamin K3, is a synthetic naphthoquinone derivative with well-documented pro-oxidant properties. It undergoes intracellular redox cycling, producing semiquinone radicals that reduce molecular oxygen to form superoxide and downstream hydrogen peroxide, ultimately leading to accumulation of ROS and oxidative stress in cancer cells (Li et al., 2019; Petricciuolo et al., 2020). Menadione-induced ROS has been associated with mitochondrial dysfunction and activation of caspase-dependent apoptotic pathways (Loor et al., 2010). Moreover, menadione contributes to intracellular glutathione (GSH) depletion and thioredoxin reductase inhibition, further impairing redox homeostasis and enhancing its cytotoxic potential (Li et al., 2019; Petricciuolo et al., 2020). Although both compounds promote ROS formation via quinone redox cycling, DOX cytotoxicity is additionally dominated by DNA intercalation and topoisomerase II poisoning, whereas menadione is commonly used as a prototypical pro-oxidant naphthoquinone whose toxicity is closely linked to redox cycling coupled with glutathione/thiol perturbation.

Therefore, differential sensitivities of EC cell lines to DOX versus menadione may not solely reflect differences in ROS generation, but also cell-line-specific determinants and additional drug activities, including DNA damage responses, topoisomerase II dependence, and drug transport/efflux mechanisms.

ROS act as double-edged regulators in cancer biology. At moderate levels, they contribute to signaling and proliferation, whereas excessive accumulation promotes apoptosis (Burdon, 1995; He et al., 2016; Kozak et al., 2020). These effects are counterbalanced by antioxidant defense systems, including enzymatic factors such as SOD and stress-inducible proteins such as SESN2, SESN3, which regulate redox homeostasis and modulate cell survival pathways (Lee et al., 2013; He et al., 2017). SESNs are stress-inducible proteins that contribute to cellular defense against oxidative and metabolic stress (Budanov et al., 2004). Their antioxidant activity involves both direct redox-related functions and indirect signaling mechanisms. Structural and functional studies indicate that SESN2 contains an oxidoreductase-like domain, and SESNs have been implicated in limiting oxidative damage, in part through regulation of peroxiredoxin redox cycling (Woo et al., 2009; Kim et al., 2015). Moreover, SESN2 and SESN3 attenuate ROS accumulation by suppressing mTORC1 signaling (via AMPK- and/or GATOR-dependent nutrient signaling), thereby promoting autophagy and restoring cellular homeostasis (Budanov and Karin, 2008; Budanov et al., 2010). SESNs can also enhance antioxidant gene expression by facilitating Nrf2 activation, for example through p62-dependent sequestration and degradation of Keap1, which derepresses Nrf2-driven transcriptional programs (Budanov and Karin, 2008; Budanov et al., 2010). Consistent with these functions, dysregulated SESN2/SESN3 expression has been reported across multiple cancer types and has been linked to altered proliferation, survival, stress adaptation, and therapy response (Kozak and Jonak, 2023).

In this study, we investigated how DOX and menadione affect oxidative stress responses in AN3CA and KLE cell lines. We focused on cell viability, ROS generation, and the regulation of antioxidant mechanisms, with particular emphasis on SESN2 and SESN3 in comparison to SOD1 as a classical enzymatic control. To our knowledge, this is the first systematic comparison of DOX- and menadione-induced oxidative stress defense in these aggressive EC models.

## Materials and methods

### Cell culture

The EC cell lines KLE (CRL-1622™) and AN3CA (HTB-111™) were obtained from the American Type Culture Collection (ATCC, Manassas, VA, United States). KLE cells were cultured in DMEM/F12 (Gibco, Thermo Fisher Scientific, Waltham, MA, United States) medium supplemented with 10% fetal bovine serum (FBS, Gibco) and 2% penicillin/streptomycin (Gibco). AN3CA cells were maintained in EMEM (Gibco) supplemented with 10% FBS and 2% penicillin/streptomycin. All cells were grown at 37 °C in a humidified atmosphere with 5% CO_2_. Cells were detached by standard trypsinization (Gibco) and counted using a Scepter™ 3.0 Cell Counter (Merck, Germany).

### ATPlite asay

Cell viability was assessed using the ATPlite Luminescence Assay System (Revvity, Waltham, MA, United States). KLE and AN3CA cells were seeded in black 96-well plates at 10,000 and 20,000 cells/well, respectively, and cultured to 80%–90% confluence. Cells were treated for 24 h with DOX (4–0.125 μM; Thermo Fisher Scientific, Waltham, MA, United States) or menadione (25–1 μM; Carl Roth, Germany). DMSO (SERVA, Germany) was used as a solvent control at concentrations equivalent to the highest and lowest doses.

After treatment, ATPlite assays were performed according to the manufacturer’s instructions. Luminescence was measured on a VICTOR Nivo™ Multimode Microplate Reader (Revvity). Each concentration was tested in six technical replicates, and all experiments were independently repeated twice.

### Measurement of ROS generation

Intracellular ROS/oxidative stress was assessed using CellROX® Orange Oxidative Stress Reagent (Thermo Fisher Scientific, Waltham, MA, United States). CellROX® Orange is a cell-permeant fluorogenic probe that is weakly fluorescent in its reduced form and becomes strongly fluorescent upon oxidation by intracellular reactive oxygen species. Thus, it reports overall intracellular oxidative stress (general ROS/oxidant burden) rather than a single defined ROS species. KLE and AN3CA cells were seeded in black 96-well plates at densities of 10,000 and 20,000 cells/well, respectively, and incubated for 24 h at 37 °C in 5% CO_2_ to reach 80%–90% confluence. Cells were then treated with DOX (0.125–4 μM; Thermo Fisher Scientific, Waltham, MA, United States) or menadione (1–25 μM; Carl Roth, Germany), with DMSO (SERVA, Germany) as the vehicle control, under the same conditions as described for the ATPlite assay. After 24 h of exposure, CellROX® Orange was added to each well to achieve a final concentration of 5 μM. Plates were incubated for 30 min at 37 °C protected from light. The medium was then aspirated, and cells were washed three times with PBS. Fluorescence was measured directly from adherent cells (cell-associated signal) using a VICTOR Nivo™ Multimode Microplate Reader (Revvity) at Ex/Em 545/565 nm. Each concentration was tested in six technical replicates, and experiments were independently repeated twice. Fluorescence was reported as RFU per well (cell-associated signal) and was not additionally normalized to protein content or cell number. Therefore, the readout was interpreted as an integrated oxidative stress measure and considered together with viability data.

### In-Cell Western assay

KLE and AN3CA cells were seeded in black 96-well plates at densities of 10,000 and 20,000 cells/well, respectively, and cultured for 24 h at 37 °C in 5% CO_2_ to reach 80%–90% confluence. Cells were treated with menadione (25, 10, 5, 1 µM) or DOX (4, 2, 1, 0.5, 0.25, 0.125 µM) for 24 h, with medium-only controls included. After treatment, cells were fixed with 4% paraformaldehyde for 20 min and permeabilized with 0.1% Triton X-100 in PBS. Blocking was performed using Intercept® Blocking Buffer (PBS).

Primary antibodies against SESN2, SESN3, and SOD1 (Sigma-Aldrich, St. Louis, MO, United States) were optimized in preliminary experiments across dilution ranges (1:100–1:1200), and optimal working concentrations (1:150, 1:100, and 1:500, respectively) were used in subsequent assays. Antibodies were diluted in Intercept® Blocking Buffer containing 0.05% Tween-20. Overnight incubation at 4 °C was followed by washing in PBS-T (0.1% Tween-20). Secondary antibodies (1:800, LI-COR) were applied together with CellTag™ 520 Stain (1:500, LI-COR) to enable normalization to cell number (in-well loading control). SESN2 and SESN3 were quantified in multiplex within the same wells using spectrally distinct secondary antibodies: SESN2 was detected with anti-rabbit secondary antibodies in the 700 nm channel, whereas SESN3 was detected with anti-mouse secondary antibodies in the 800 nm channel; CellTag™ 520 signal was acquired in the 520 nm channel. SOD1 was analyzed in a separate In-Cell Western (ICW) experiment due to limited available detection channels when using CellTag™ 520 for normalization; in this assay, SOD1 was detected with anti-rabbit secondary antibodies in the 700 nm channel, with CellTag™ 520 acquired in the 520 nm channel.

Fluorescence signals were detected using the Odyssey® M Imaging System (LI-COR, Lincoln, NE, United States) in the 700, 800, and 520 nm channels. Background controls included wells incubated without primary antibodies. Signal acquisition, quantification, and normalization were performed using Empiria Studio® software (LI-COR). For quantification, target-specific fluorescence was expressed as target/CellTag™ 520 ratios obtained from the same wells to correct for well-to-well variation in cell number (normalized signal). Untreated control (medium-only) wells were included in each experiment and served as the reference group for comparisons.

Replication and experimental repeats. For doxorubicin, the ICW assay was performed in two independent runs on different days. In the first run, each DOX concentration was measured in three technical replicate wells, and in the second run in six technical replicate wells (all other experimental conditions were identical). For menadione, the ICW assay was performed in one run, with six technical replicate wells per concentration, under the same assay conditions as used for DOX. Data are presented as mean ± SD of technical replicates for each run; for DOX, the observed effects were consistent across both independent runs.

### Extraction and quality assessment of RNA

KLE and AN3CA cells were seeded in 6-well plates (4 × 10^5^ and 8 × 10^5^ cells/well, respectively) and treated for 24 h with DOX (2 and 4 μM; Thermo Fisher Scientific) or menadione (10 and 25 μM; Carl Roth, Germany These concentrations were selected based on their statistically significant effects on ROS generation in AN3CA and KLE cells. Medium-only controls were included. Following treatment, cells were harvested, and total RNA was extracted using the Total RNA Mini Plus kit (A&A Biotechnology, Gdansk, Poland) according to the manufacturer’s protocol. Approximately 1 × 10^6^ cells were used per isolation.

RNA concentration and purity were assessed with NanoDrop™ One (Thermo Fisher Scientific), and all samples showed 260/280 ratios between 1.8 and 2.2. RNA integrity was evaluated with the Agilent TapeStation 4150 using RNA ScreenTape (Agilent Technologies Inc., Santa Clara, CA, United States), and only samples with RNA integrity number (RIN) ≥ 9 (range 9–10) were used for downstream analyses. RNA was stored at −80 °C until use.

### Quantitative real-time polymerase chain reaction (qRT-PCR) analysis of total RNA

Total RNA (500 ng) was reverse transcribed using the iScript cDNA Synthesis Kit (Bio-Rad, Hercules, CA, United States) following the manufacturer’s protocol. The resulting cDNA was stored at −20 °C until use.

Quantitative PCR was performed in technical triplicate using the iTaq Universal Probes Supermix (Bio-Rad) and the CFX Opus Real-Time PCR detection system (Bio-Rad). PrimePCR™ Probe Assay was used for SOD1 (ID: qHsaCIP0026883, Bio-Rad), while TaqMan® Gene Expression Assays were used for SESN2 (ID: Hs00230241_m1), SESN3 (ID: Hs00914870_m1), and UBC (ID: Hs00824723_m1) (Thermo Fisher Scientific, Waltham, MA, United States). Cycling conditions were 95 °C for 10 s, followed by 45 cycles of 95 °C for 3 s and 60 °C for 30 s. Relative mRNA expression was calculated using the 2^–ΔΔCt method, with UBC as the endogenous reference gene and untreated control (medium-only) samples as the calibrator. Key findings were confirmed in an independent repeat experiment.

### Data analysis

Statistical analyses were performed using Microsoft Excel and GraphPad Prism (version 10). Data are presented as mean ± SD. For comparisons across multiple groups, ordinary one-way ANOVA was conducted, followed by Dunnett’s *post hoc* test to compare each treatment condition with the untreated control (medium-only). For antibody optimization experiments, differences were evaluated using two-way ANOVA (cell line × antibody dilution) followed by Sidak’s multiple-comparisons test. For ICW experiments, signal quantification and normalization were performed in Empiria Studio® (LI-COR) prior to statistical testing in GraphPad Prism. A p-value <0.05 was considered statistically significant.

## Results

### The impact of doxorubicin and menadione on cellular viability

**FIGURE 1:**
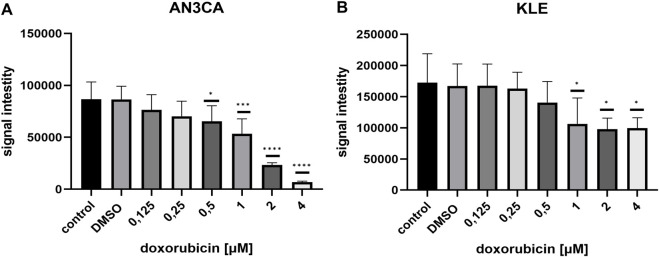
ATP-based viability assays under DOX treatment. **(A)** AN3CA cells were exposed to increasing DOX concentrations (0.125–4 µM). The ATPlite assay revealed a dose-dependent reduction in viability, evident from 0.5 µM. **(B)** KLE cells treated with DOX (0.125–4 µM) displayed a moderate but non–dose-dependent reduction in viability. Data are shown as mean ± SD of six technical replicate wells per condition. Statistical significance was determined by one-way ANOVA followed by Dunnett’s *post hoc* test comparing each treatment condition to the untreated control (medium-only). Vehicle control (DMSO) was included and showed no significant difference vs. untreated control. Experiments were repeated independently twice with consistent results. *p < 0.05; ***p < 0.0005 ****p < 0.0001.

The effects of DOX and menadione on cell viability were evaluated using an ATP-based luminescence assay. ATP serves as a marker of metabolically active cells, and its depletion reflects necrosis or apoptosis. The intensity of emitted light is directly proportional to the intracellular ATP concentration. DOX induced a dose-dependent reduction in cell viability for both AN3CA and KLE cell lines (Figures 1A,B). In AN3CA cells, significant reductions were observed at 0.5 μM (24.45% ± 17.26; n = 6; p < 0.05), 1 μM (38.31% ± 16.59; n = 6; p < 0.05), 2 μM (72.35% ± 2.58; n = 6; p < 0.0001), and 4 μM (91.81% ± 1.61; n = 6; p < 0.0001) compared with control. In KLE cells, DOX produced a moderate but non-dose-dependent reduction in viability: 38.35% ± 24.00 (n = 4; p < 0.05) at 1 μM, 43.3% ± 10.0 (n = 4; p < 0.05) at 2 μM, and 42.27% ± 9.66 (n = 3; p < 0.05) at 4 μM versus control.

**FIGURE 2:**
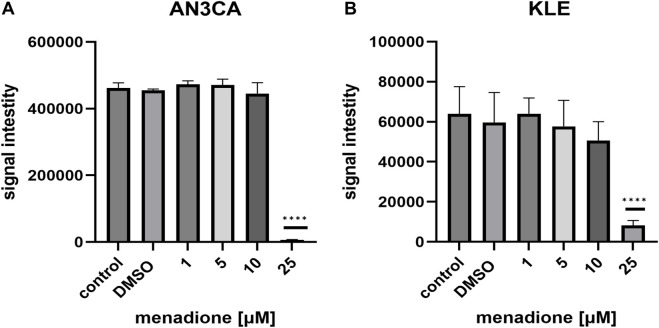
ATP-based viability assays under menadione treatment. **(A)** AN3CA cells were exposed to menadione (1–25 µM). The ATPlite assay showed strong cytotoxic effects at 25 µM. **(B)** KLE cells treated with menadione (1–25 µM) also exhibited markedly reduced viability at 25 µM. Data are shown as mean ± SD of six technical replicate wells per condition. Statistical significance was determined by one-way ANOVA followed by Dunnett’s *post hoc* test comparing each treatment condition to the untreated control (medium-only). Vehicle control (DMSO) was included and showed no significant difference vs. untreated control. Experiments were repeated independently twice with consistent results. ****p < 0.0001.

Menadione exhibited strong cytotoxic effects at higher concentrations. In AN3CA cells, only 25 μM caused a significant decrease in viability (98.63% ± 0.31; n = 7; p < 0.0001) (Figure 2A). In KLE cells, 25 μM also reduced viability markedly (87.0% ± 3.73; n = 8; p < 0.0001) (Figure 2B). Together, these results indicate that AN3CA cells are more susceptible to DOX than KLE cells, while both lines show high sensitivity to 25 μM menadione, with AN3CA displaying near-complete loss of viability.

### The influence of doxorubicin and menadione treatment on reactive oxygen species production

**FIGURE 3:**
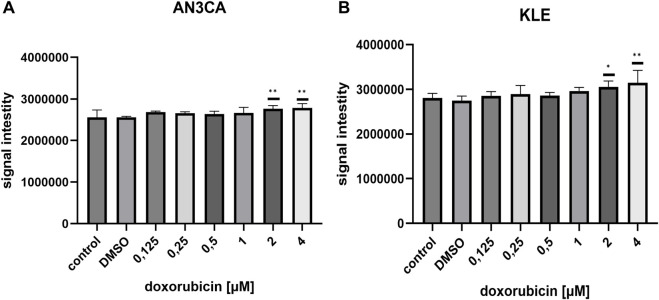
ROS generation induced by DOX in EC cell lines. **(A)** AN3CA cells were exposed to DOX (0.125–4 µM) for 24 h. ROS levels, measured using the CellROX® assay, were significantly elevated at 2 μM and 4 µM. **(B)** KLE cells treated under the same conditions showed a slight increase in ROS at 2 μM and 4 µM. Data are shown as mean ± SD of six technical replicate wells per condition. Statistical significance was determined by one-way ANOVA followed by Dunnett’s *post hoc* test comparing each treatment condition to the untreated control (medium-only). Vehicle control (DMSO) was included and showed no significant difference vs. untreated control. Experiments were repeated independently twice with consistent results. *p < 0.05; **p < 0.005.

Because both DOX and menadione are known ROS-inducing agents, we evaluated their ability to promote ROS production in AN3CA and KLE cells using CellROX staining. In AN3CA cells, DOX significantly increased ROS levels at 2 μM (1.08 ± 0.03-fold vs. control; n = 6; p < 0.01) and 4 μM (1.08 ± 0.04-fold; n = 6; p < 0.01) (Figure 3A). In contrast, ROS induction by DOX in KLE cells was modest, with a slight increase at 2 μM (1.08 ± 0.04-fold; n = 6; p < 0.05) and 4 μM (1.12 ± 0.09-fold; n = 6; p < 0.01) (Figure 3B). Lower concentrations of DOX (0.125–1 μM) did not significantly alter ROS levels in either cell line, suggesting effective antioxidant defense at subtoxic doses.

**FIGURE 4:**
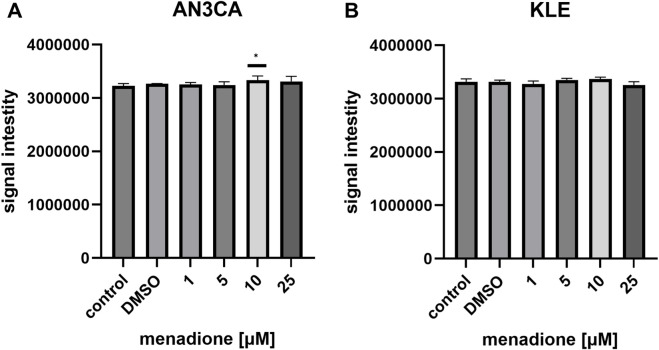
ROS generation induced by menadione in EC cell lines. **(A)** AN3CA cells treated with menadione (1–25 µM) for 24 h displayed a significant ROS increase at 10 µM. **(B)** In KLE cells, ROS elevation remained marginal and did not reach statistical significance across tested doses. Data are shown as mean ± SD of six technical replicate wells per condition. Statistical significance was determined by one-way ANOVA followed by Dunnett’s *post hoc* test comparing each treatment condition to the untreated control (medium-only). Vehicle control (DMSO) was included and showed no significant difference vs. untreated control. Experiments were repeated independently twice with consistent results. *p < 0.05.

Menadione induced ROS in AN3CA cells, with a significant increase observed at 10 μM (1.03 ± 0.02-fold vs. control; n = 6; p < 0.05) (Figure 4A), whereas in KLE cells ROS elevation remained marginal and did not reach statistical significance at the tested doses (Figure 4B). These findings may reflect cell line–specific redox homeostasis, and it cannot be excluded that ROS generation by menadione in KLE cells occurs with different kinetics than those captured at the chosen 24 h timepoint.

### Utilization of the In-Cell Western technique for monitoring protein levels in the EC cell line

To quantify SESN2, SESN3, and SOD1 protein levels, we optimized and applied the In-Cell Western (ICW) technique, a plate-based immunofluorescence assay that allows high-throughput analysis directly in fixed cells. This approach circumvents protein extraction and electrophoresis required in conventional Western blotting, thereby increasing reproducibility and reducing variability. A critical step in establishing the assay was the validation of primary antibodies for compatibility with immunofluorescence.

**FIGURE 5:**
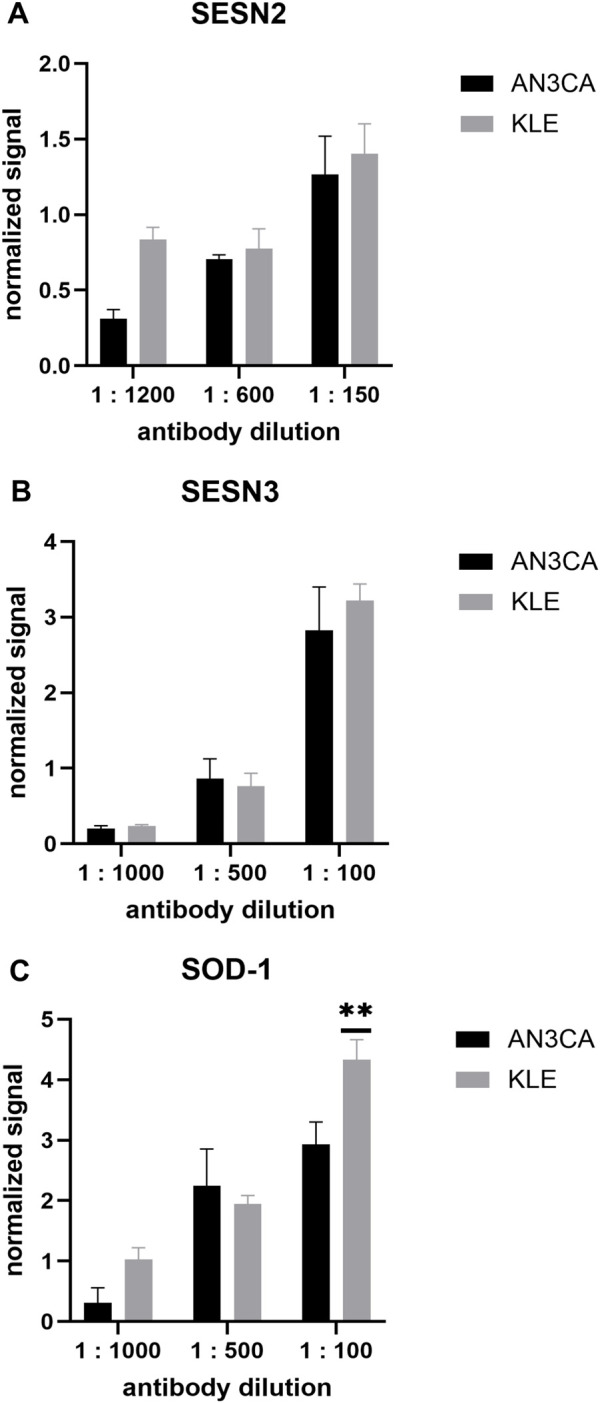
Validation of primary antibodies against SESN2, SESN3, and SOD1 using ICW assay. **(A)** AN3CA and KLE cells were probed with anti-SESN2 antibody (dilutions 1:1200–1:150). **(B)** AN3CA and KLE cells were probed with anti-SESN3 antibody (dilutions 1:1000–1:100). **(C)** AN3CA and KLE cells were probed with anti-SOD1 antibody (dilutions 1:1000–1:100). Secondary antibodies (1:800, LI-COR) were applied, with CellTag™ 520 (1:500, LI-COR) as a loading control for signal normalization. Data are shown as mean ± SD of three technical replicate wells from a single antibody-optimization experiment. Statistical significance was assessed by two-way ANOVA (factors: cell line and antibody dilution), followed by Sidak’s multiple-comparisons test comparing AN3CA vs. KLE within each dilution. **p < 0.005.

Optimization experiments demonstrated that the highest normalized signal for SESN2 was obtained with a 1:150 dilution (AN3CA 1.26 ± 0.25; KLE 1.4 ± 0.19) (Figure 5A), while SESN3 and SOD1 were optimally detected at 1:100 (SESN3: AN3CA 2.82 ± 0.5; KLE 3.21 ± 0.21 SOD1: AN3CA 2.93 ± 0.36; KLE 4.33 ± 0.32) (Figures 5B,C). Using these conditions, baseline SESN2 and SESN3 expression appeared slightly higher in untreated KLE cells than in AN3CA cells, although these differences did not reach statistical significance (p > 0.05). By contrast, SOD1 protein levels were significantly higher in untreated KLE cells compared with AN3CA (4.33 ± 0.32 vs. 2.93 ± 0.36; n = 3; p = 0.0043) (Figure 5C). These results establish ICW as a robust and reproducible method for monitoring treatment-induced changes in antioxidant protein expression in endometrial carcinoma cells.

### In cell western analysis of SOD1, SESN2, SESN3 level in AN3CA and KLE EC cells under menadione and doxorubicin treatment

The resistance of tumors to cytotoxic agents may be linked to the heightened expression of endogenous antioxidant proteins within cancer cells. In order to investigate the protective function of these antioxidant proteins during exposure to menadione and DOX in EC cell lines KLE and AN3CA, SOD1 was chosen as a representative marker for antioxidant proteins, while SESN2 and SESN3 proteins were identified as novel potential players in antioxidative activity.

### SOD1 protein level in AN3CA and KLE cells after menadione exposure

**FIGURE 6:**
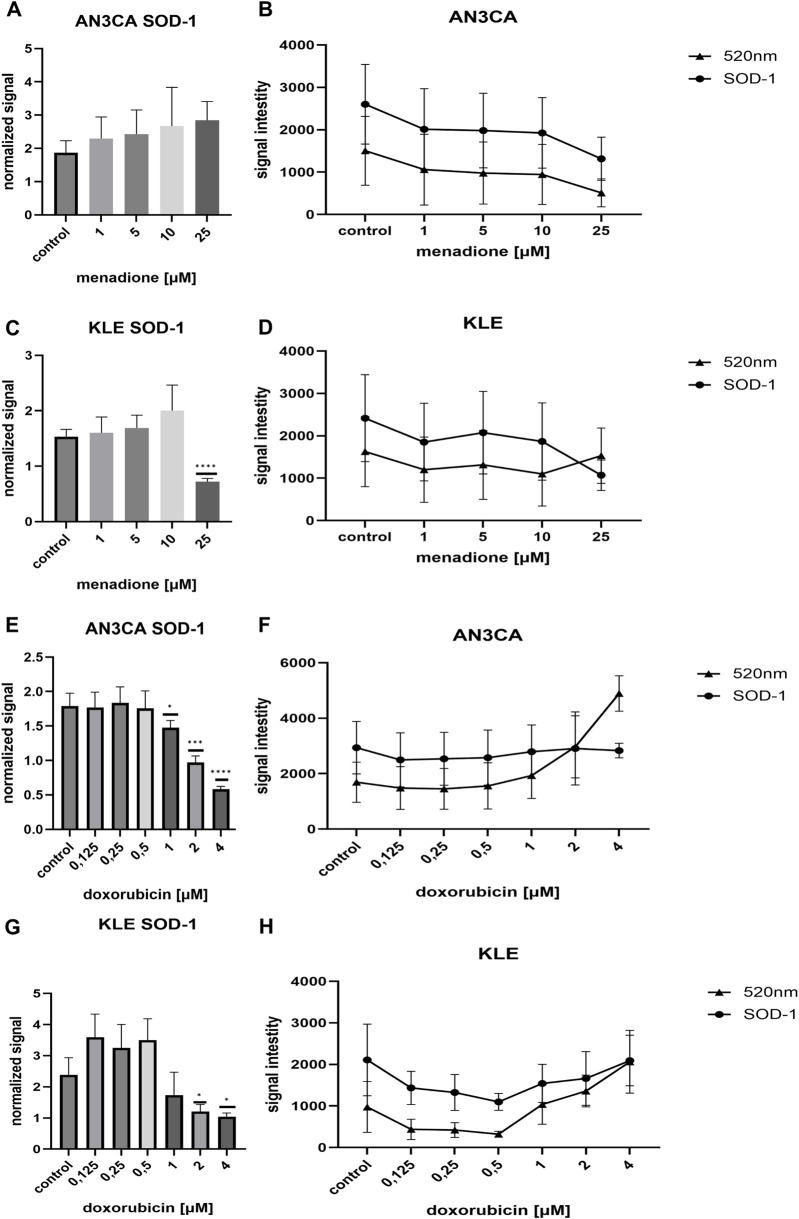
Differential effects of DOX and menadione on SOD1 expression in AN3CA and KLE cells assessed by ICW assay. **(A)** In AN3CA cells, menadione (1–25 µM) induced an upward trend in normalized SOD1 levels. **(B)** Raw fluorescence signals corresponding to SOD1 (700 nm) and CellTag™ 520 (520 nm). **(C)** In KLE cells, menadione increased normalized SOD1 levels at 1–10 μM, followed by a marked decrease at 25 µM. **(D)** Raw fluorescence signals corresponding to SOD1 (700 nm) and CellTag™ 520 (520 nm). **(E)** In AN3CA cells treated with DOX (0.125–4 µM), normalized SOD1 levels remained relatively stable at lower concentrations (0.25–0.5 µM) but declined markedly from 1 µM onward. **(F)** Raw fluorescence signals corresponding to SOD1 (700 nm) and CellTag™ 520 (520 nm). **(G)** In KLE cells, DOX induced dose-dependent modulation of SOD1 protein levels. **(H)** Raw fluorescence signals corresponding to SOD1 (700 nm) and CellTag™ 520 (520 nm). Normalized protein levels were calculated as SOD1/CellTag™ 520 ratios (normalized signal). Untreated control (medium-only) wells were included in each experiment and served as the reference group for comparisons. Statistical significance was determined by one-way ANOVA followed by Dunnett’s *post hoc* test comparing each treatment condition to the untreated control. For DOX, the figure shows a representative ICW run (n = 6 technical replicate wells per condition), and the experiment was repeated independently once with consistent results (n = 3). For menadione, data are from one ICW experiment (n = 6 technical replicate wells per condition). *p < 0.05; ***p < 0.0005; ****p < 0.0001.

To assess the antioxidant response in AN3CA EC cells, SOD1 protein levels were quantified following exposure to increasing concentrations of menadione (1–25 µM) using ICW analysis. Normalized SOD1 signal showed an apparent upward trend with rising menadione concentrations, from 1.87 ± 0.35 in untreated controls to 2.84 ± 0.56 at 25 µM (Figure 6A). However, statistical analysis via one-way ANOVA indicated that these changes were not significant (F = 1.351, p = 0.2844), suggesting high inter-replicate variability. While the data hint at a concentration-dependent induction of SOD1, this trend requires further validation.

To support the normalized ICW findings, raw fluorescence intensities from the 700 nm (SOD1 detection) and 520 nm (CellTag 520 normalization) channels were analyzed (Figure 6B). CellTag 520 served to correct for potential differences in cell density, enabling more accurate per-cell quantification. Signal stability in the 520 nm channel across concentrations supports the integrity of normalization, though biological variability limits definitive conclusions. These findings underline the need for complementary assays to confirm the functional relevance of SOD1 modulation under oxidative stress.

In KLE EC cells, SOD1 levels exhibited a biphasic response to menadione exposure. At lower concentrations (1–10 µM), normalized SOD1 signal increased modestly from 1.53 ± 0.13 in controls to 1.92 ± 0.46 at 10 μM, consistent with an adaptive antioxidant response (Figure 6C). However, at 25 µM menadione, a marked decrease was observed (0.71 ± 0.05), suggesting either cytotoxic stress or downregulation of SOD1 under excessive oxidative burden. One-way ANOVA confirmed significant differences across groups (F = 18.4, p < 0.0001), with the most pronounced change being the suppression of SOD1 at 25 µM.

Raw fluorescence analysis further supported this interpretation (Figure 6D). At 1–10 μM, 700 nm signal intensities increased slightly while 520 nm (CellTag 520) signals remained stable, indicating maintained cell numbers. In contrast, 25 µM menadione led to a significant reduction in the 700 nm signal accompanied by an unexpected rise in the 520 nm signal. This pattern may reflect morphological changes such as chromatin condensation during apoptosis, which could artificially elevate the normalization signal.

These results emphasize the importance of interpreting normalized ICW data with caution under conditions of high cytotoxicity. Alterations in cell morphology may confound per-cell quantification, potentially leading to underestimation of protein expression levels.

### SOD1 protein level in AN3CA and KLE cells after doxorubicin exposure

DOX exposure led to a progressive decline in SOD1 protein levels in AN3CA cells, as assessed by normalized ICW signal intensities (Figure 6E). At lower doses (0.25–0.5 µM), SOD1 expression remained relatively stable (1.76 ± 0.22 to 1.75 ± 0.25), suggesting maintenance of basal antioxidant responses. However, starting from 1 μM, a marked reduction was observed, with SOD1 levels dropping to 0.58 ± 0.04 at 4 μM, compared to 1.78 ± 0.18 in untreated controls. One-way ANOVA revealed statistically significant differences across treatment conditions (F = 40.83, p < 0.0001), confirming DOX-induced suppression of SOD1 at cytotoxic concentrations.

Analysis of raw fluorescence intensities at 700 nm (SOD1 detection) and 520 nm (CellTag 520 normalization) further contextualized these findings (Figure 6F). While the 700 nm signal remained relatively stable, the 520 nm signal increased notably at 2–4 µM DOX, possibly due to apoptosis-related chromatin condensation or morphological changes that artificially amplify the normalization signal. This phenomenon may lead to an underestimation of per-cell SOD1 levels when relying solely on normalized data.

These observations suggest that DOX suppresses SOD1 expression in a dose-dependent manner in AN3CA cells, potentially reflecting oxidative stress that exceeds cellular antioxidant capacity. Caution is warranted when interpreting normalized ICW results under conditions of extensive cytotoxicity, as signal distortions may arise from apoptotic cell morphology.

In KLE cells, ICW analysis revealed a dose-dependent modulation of SOD1 protein levels in response to DOX treatment (Figure 6G). At low concentrations (0.125–0.5 µM), SOD1 expression increased relative to control (2.38 ± 0.55), reaching values of 3.59 ± 0.74, 3.25 ± 0.74, and 3.49 ± 0.68, respectively. This likely reflects an early antioxidant response to mild oxidative stress. However, at concentrations ≥1.0 µM, SOD1 levels declined progressively, reaching 1.04 ± 0.11 at 4 µM. One-way ANOVA confirmed that this downregulation was statistically significant (F = 15.77, p < 0.0001), particularly at 2 μM and 4 µM.

Raw fluorescence data further clarified this trend (Figure 6H). While 700 nm signal intensities remained relatively high at 1–4 μM, the concurrent increase in CellTag 520 signal suggests possible changes in cell morphology or biomass—such as chromatin condensation, cell cycle arrest, or hypertrophy—rather than simple loss of viability. This elevated normalization signal may obscure true protein abundance per cell and warrants careful interpretation.

These results suggest that KLE cells initially mount a SOD1–mediated protective response to DOX, but at higher doses, antioxidant defenses may become overwhelmed or repressed. Notably, this response differs from AN3CA cells, where SOD1 levels were sharply reduced under similar conditions. The divergence underscores cell line–specific redox dynamics in response to chemotherapeutic stress and may hold therapeutic relevance for tailoring redox-based interventions.

### SESN2 protein level in AN3CA and KLE cell lines after menadione exposure

SESN2 protein levels were assessed in AN3CA and KLE EC cell lines using ICW analysis following 24-h exposure to increasing concentrations of menadione (1, 5, 10, and 25 µM). Fluorescence signals were recorded at 700 nm and normalized using CellTag 520 at 520 nm to account for cell number variations.

**FIGURE 7:**
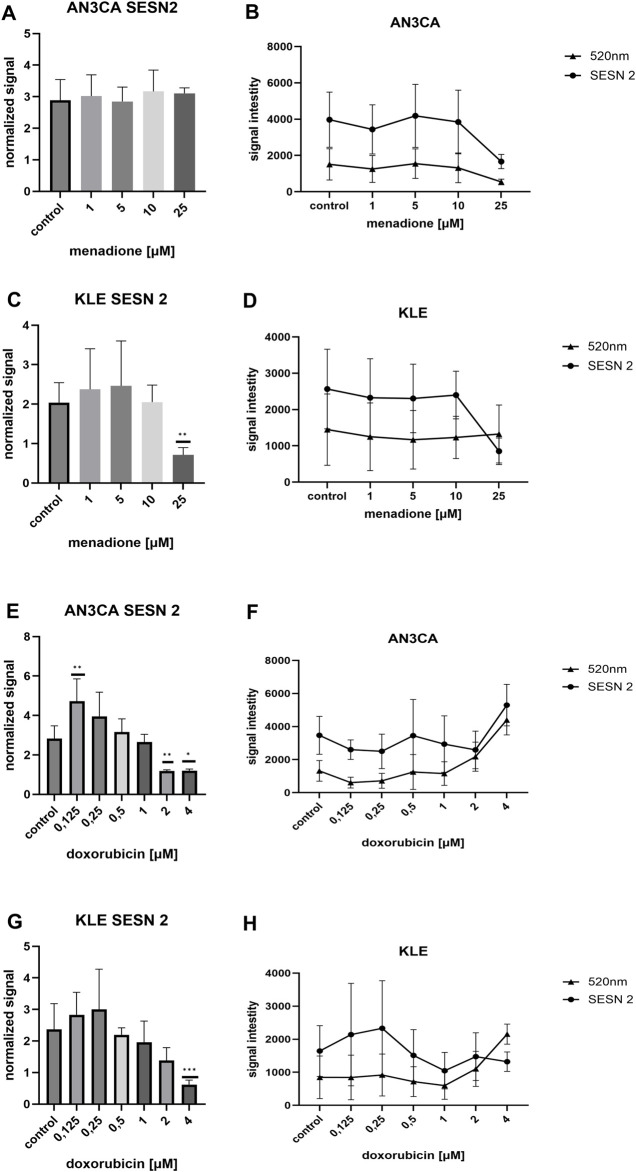
Differential effects of DOX and menadione on SESN2 expression in AN3CA and KLE cells assessed by ICW assay. **(A)** In AN3CA cells, menadione (1–25 µM) induced a modest upward trend in normalized SESN2 levels. **(B)** Raw fluorescence signals corresponding to SESN2 (700 nm) and CellTag™ 520 (520 nm). **(C)** In KLE cells, menadione increased normalized SESN2 levels at 1–5 μM, followed by a marked decrease at 25 µM. **(D)** Raw fluorescence signals corresponding to SESN2 (700 nm) and CellTag™ 520 (520 nm). **(E)** In AN3CA cells treated with DOX (0.125–4 µM), SESN2 exhibited a biphasic response, peaking at 0.125 µM and declining sharply from 1 µM onward. **(F)** Raw fluorescence signals corresponding to SESN2 (700 nm) and CellTag™ 520 (520 nm). **(G)** In KLE cells, DOX induced modest SESN2 upregulation at 0.125–0.25 µM, followed by progressive downregulation with the lowest levels at 4 µM. **(H)** Raw fluorescence signals corresponding to SESN2 (700 nm) and CellTag™ 520 (520 nm). Normalized protein levels were calculated as SESN2/CellTag™ 520 ratios (normalized signal). Untreated control (medium-only) wells were included in each experiment and served as the reference group for comparisons. Statistical significance was determined by one-way ANOVA followed by Dunnett’s *post hoc* test comparing each treatment condition to the untreated control. For DOX, the figure shows a representative ICW run (n = 6 technical replicate wells per condition), and the experiment was repeated independently once with consistent results (n = 3). For menadione, data are from one ICW experiment (n = 6 technical replicate wells per condition). *p < 0.05; **p < 0.005; ***p < 0.0005.

In AN3CA cells, SESN2 expression showed a modest, dose-dependent increase, rising from 2.88 ± 0.65 in untreated controls to 3.17 ± 0.67 at 10 μM and 3.10 ± 0.17 at 25 µM (Figure 7A). A slight dip at 5 µM (2.84 ± 0.45) was observed, though this may reflect technical variation rather than biological significance. The overall trend suggests mild induction of SESN2 under oxidative stress.

In contrast, KLE cells exhibited a distinct response, with SESN2 levels peaking at 5 µM (2.46 ± 1.14) before declining sharply at 25 µM (0.71 ± 0.18), as shown in Figure 7C. This pronounced reduction may indicate either cytotoxic saturation or negative feedback regulation at higher oxidative loads. Raw fluorescence data (700 nm) corroborated normalized trends in both cell lines, and 520 nm normalization signals remained stable across doses (Figures 7B,D), supporting data integrity.

These findings highlight differential SESN2 regulation in response to redox stress between the two EC cell lines. While AN3CA cells maintain stable SESN2 induction, KLE cells appear more sensitive to oxidative overload, suggesting a lower threshold for stress tolerance. This supports a possible role for SESN2 in mediating cell line–specific antioxidant responses.

### SESN2 protein level in AN3CA and KLE cell lines following doxorubicin exposure

SESN2 protein levels were quantified in AN3CA and KLE EC cells using ICW following 24-h exposure to DOX at increasing concentrations (0.125–4.0 µM). Fluorescence at 700 nm was normalized to CellTag 520 signal (520 nm) to account for cell density variability.

In AN3CA cells, SESN2 exhibited a biphasic response (Figure 7E). A peak in normalized signal (4.72 ± 1.13) was observed at 0.125 µM, suggesting strong early induction under mild oxidative stress. However, at concentrations ≥1.0 µM, SESN2 levels declined sharply, likely reflecting either suppression of SESN2 transcription or compromised cell viability at higher cytotoxic stress levels.

In contrast, KLE cells showed a more gradual response (Figure 7G). SESN2 levels increased modestly at 0.125–0.25 µM (2.83 ± 0.71–3.00 ± 1.27), followed by progressive downregulation at higher concentrations, with the lowest value at 4.0 µM (0.61 ± 0.13). This indicates a potentially lower threshold for oxidative stress adaptation compared to AN3CA.

Interestingly, raw 700 nm and 520 nm fluorescence signals did not always mirror normalized trends (Figures 7F,H). At high DOX concentrations (2–4 µM), the 520 nm signal increased substantially in both cell lines, possibly due to chromatin condensation or apoptotic morphology that artificially inflates the normalization factor. Notably, 700 nm signal also increased at these doses, particularly in AN3CA cells, suggesting either SESN2 accumulation in non-viable cells or normalization artefacts.

These data highlight a dose- and cell line–dependent regulation of SESN2 in response to DOX. AN3CA cells display a sharper induction–repression profile, while KLE cells show a more attenuated, gradual decline. Differences in redox adaptation between these cell types may underlie their distinct responses to chemotherapeutic stress.

### SESN3 protein level in AN3CA and KLE cell lines after menadione exposure

SESN3 protein levels were quantified in AN3CA and KLE EC cell lines using ICW following 24-h exposure to increasing concentrations of menadione (1–25 µM). Fluorescence was recorded at 800 nm and normalized using CellTag 520 (520 nm) to control for differences in cell density.

**FIGURE 8:**
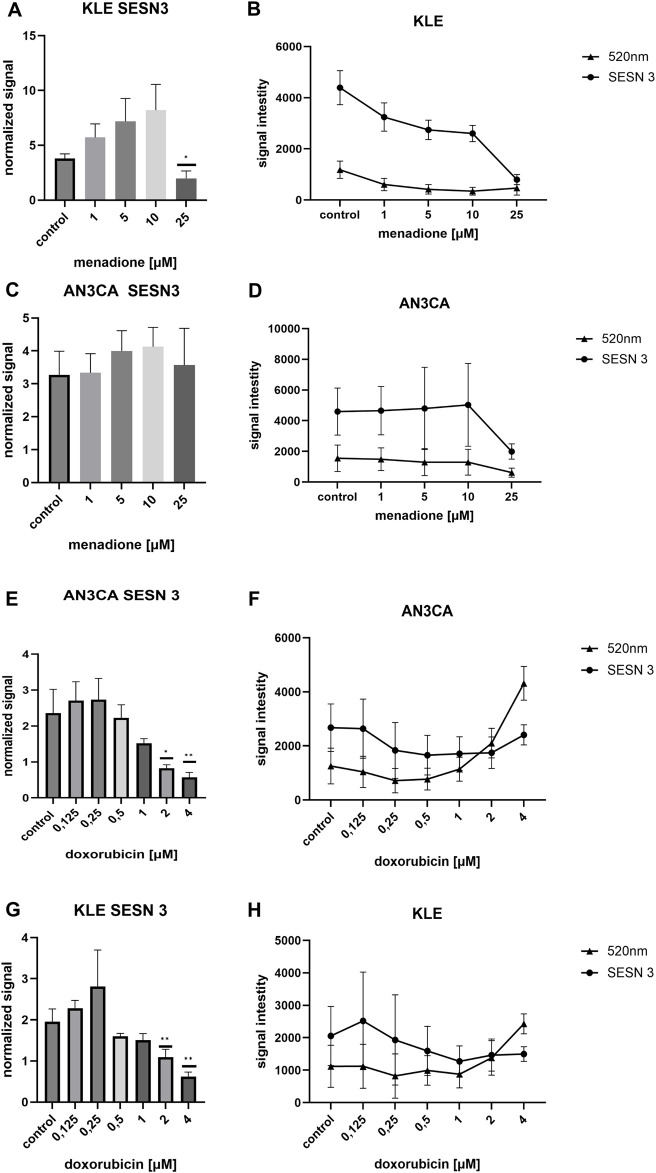
Differential effects of DOX and menadione on SESN3 expression in AN3CA and KLE cells assessed by ICW assay. **(A)** In KLE cells, menadione (1–25 µM) strongly induced SESN3 in a dose-dependent manner at 1–10 μM, followed by a marked decrease at 25 µM. **(B)** Raw fluorescence signals corresponding to SESN3 (800 nm) and CellTag™ 520 (520 nm). **(C)** In AN3CA cells, menadione produced a modest and stable SESN3 response without reaching statistical significance. **(D)** Raw fluorescence signals corresponding to SESN3 (800 nm) and CellTag™ 520 (520 nm). **(E)** In AN3CA cells exposed to DOX (0.125–4 µM), SESN3 exhibited a biphasic response, peaking at 0.25 µM and declining sharply from 0.5 µM onward. **(F)** Raw fluorescence signals corresponding to SESN3 (800 nm) and CellTag™ 520 (520 nm). **(G)** In KLE cells, DOX induced modest SESN3 upregulation at 0.125–0.25 µM, followed by gradual reduction at higher concentrations. **(H)** Raw fluorescence signals corresponding to SESN3 (800 nm) and CellTag™ 520 (520 nm). Normalized protein levels were calculated as SESN3/CellTag™ 520 ratios (normalized signal). Untreated control (medium-only) wells were included in each experiment and served as the reference group for comparisons. Statistical significance was determined by one-way ANOVA followed by Dunnett’s *post hoc* test comparing each treatment condition to the untreated control. For DOX, the figure shows a representative ICW run (n = 6 technical replicate wells per condition), and the experiment was repeated independently once with consistent results (n = 3). For menadione, data are from one ICW experiment (n = 6 technical replicate wells per condition). *p < 0.05; **p < 0.005.

In KLE cells, SESN3 expression exhibited a strong dose-dependent induction at moderate concentrations of menadione (Figure 8A). Normalized levels rose from 5.71 ± 1.24 at 1 μM to 7.18 ± 2.08 at 5 μM, peaking at 8.20 ± 2.35 at 10 µM. Despite high variability preventing statistical significance across all groups (ANOVA p > 0.05), SESN3 was significantly downregulated at 25 µM (F = 9.51, p < 0.008), indicating that excessive oxidative stress may suppress SESN3 expression or exceed the cell’s induction capacity. Raw fluorescence signals at 800 nm and 520 nm were consistent with the normalized data (Figure 8B), reinforcing the reliability of the observed trends.

In contrast, AN3CA cells demonstrated a more modest and stable SESN3 response (Figure 8C). Expression increased slightly at 1 µM (3.33 ± 0.58) and plateaued at 5–10 µM (up to 4.12 ± 0.58), followed by a mild reduction at 25 µM (3.56 ± 1.11). ANOVA revealed no statistically significant differences (F = 1.3, p = 0.31). Raw data (Figure 8D) confirmed the normalized trends and supported the validity of normalization across conditions.

These results suggest that SESN3 is inducible by redox stress in both EC cell lines but with differing sensitivity. KLE cells exhibit a pronounced, concentration-dependent induction up to a threshold, beyond which expression collapses, possibly reflecting redox exhaustion or negative feedback. AN3CA cells, in contrast, display a limited and more tightly regulated SESN3 response. These differences may reflect underlying variations in redox signaling pathways or stress tolerance between EC subtypes.

### SESN3 protein level in AN3CA and KLE cell lines after doxorubicin exposure

SESN3 protein expression was evaluated in AN3CA and KLE EC cells following 24-h exposure to increasing concentrations of doxorubicin (0.125–4.0 µM) using ICW. Normalized data indicated broadly similar expression trends in both cell lines, though with notable differences in response magnitude and dynamics.

In AN3CA cells, SESN3 levels increased modestly at low DOX concentrations (2.70 ± 0.52 at 0.125 µM and 2.73 ± 0.59 at 0.25 µM) compared to control (2.36 ± 0.65), followed by a progressive decline from 0.5 µM onward, reaching a minimum of 0.57 ± 0.13 at 4.0 µM (Figure 8E). This inverse dose-response suggests suppression of SESN3 under increasing cytotoxic stress, potentially due to transcriptional repression or apoptotic signaling. Interestingly, raw 800 nm signal initially decreased, then rose slightly at 4 µM (Figure 8F), while the 520 nm normalization signal increased sharply at 2–4 µM—possibly reflecting chromatin condensation or morphological changes associated with apoptosis or cell cycle arrest.

In KLE cells, SESN3 expression followed a similar trend but with a less steep decline (Figure 8G). Modest upregulation was observed at 0.125–0.25 µM (2.28 ± 0.18 to 2.80 ± 0.89), followed by a gradual reduction at higher doses. Unlike AN3CA, the raw 800 nm signal plateaued rather than declining at 4 µM (Figure 8H), potentially indicating stabilization of residual SESN3 protein or technical variation in signal detection.

Collectively, these data suggest that SESN3 is negatively regulated by doxorubicin in a concentration-dependent manner in both EC cell lines. However, AN3CA cells show a more rapid and pronounced suppression, indicating a potential differential sensitivity of SESN3-related pathways. This may reflect underlying differences in redox homeostasis or DNA damage response mechanisms between the two EC subtypes.

### qRT-PCR- based analysis of differential SESN2, SESN3, and SOD1 expression in endometrial cancer cells treated with doxorubicin and menadione

**FIGURE 9:**
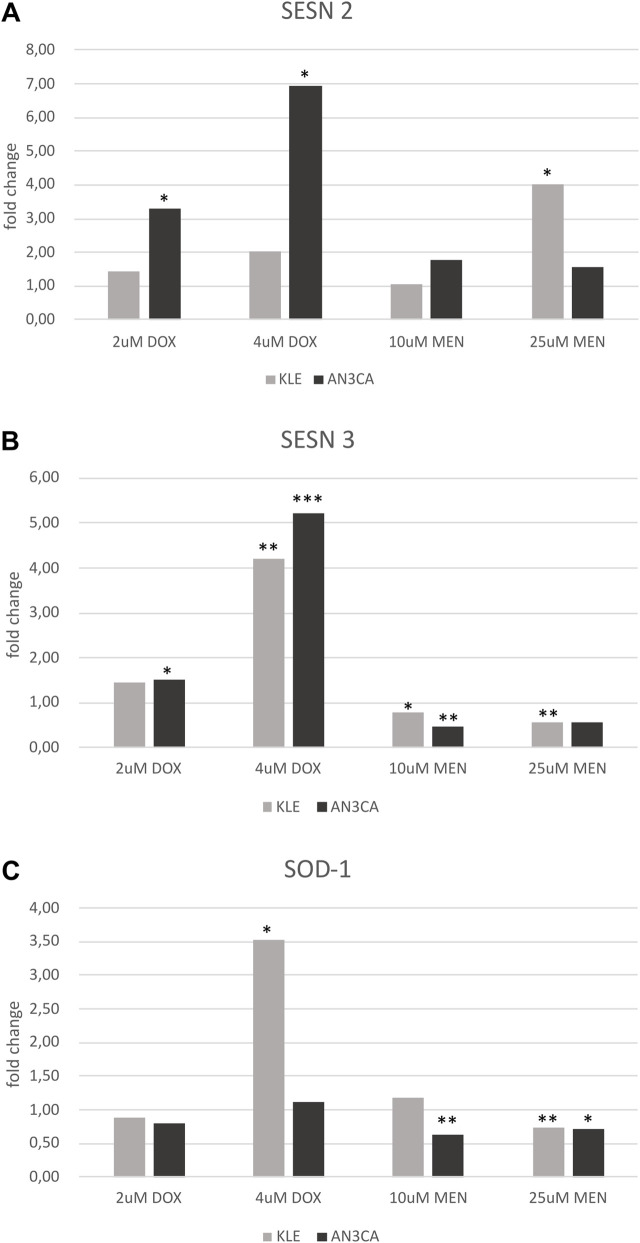
SESN2, SESN3, and SOD1 gene expression profiles in AN3CA and KLE cells analyzed by qRT-PCR. **(A)** SESN2 mRNA was significantly upregulated in both cell lines following 4 µM DOX treatment. Menadione induced robust SESN2 upregulation at 25 µM in KLE cells. **(B)** SESN3 mRNA was strongly upregulated by DOX in both lines, whereas menadione downregulated SESN3 in both cell lines. **(C)** SOD1 mRNA showed significant upregulation in KLE cells under 4 µM DOX, while AN3CA cells exhibited no significant changes. Under menadione, AN3CA cells displayed significant downregulation of SOD1 at 10 µM and 25 μM, while KLE cells showed downregulation at 25 µM. RT–qPCR data were normalized to UBC as the endogenous reference gene and are presented relative to the untreated control (medium-only). Each condition was analyzed in three technical replicate wells. Statistical significance was determined by one-way ANOVA followed by Dunnett’s *post hoc* test comparing each treatment condition to the untreated control. Key findings were confirmed in an independent repeat experiment. *p < 0.05; **p < 0.005; ***p < 0.0005.

SESN2 was significantly upregulated in both cell lines following DOX treatment (Figure 9A), with the most pronounced induction observed in AN3CA cells. At 4 µM DOX, SESN2 transcript levels increased nearly sevenfold relative to control (p < 0.01), compared to a 2.0-fold increase in KLE cells. Menadione elicited a moderate induction of SESN2 in AN3CA (1.8-fold at 10 µM), while KLE cells exhibited a more robust and statistically significant increase at 25 µM (4.0-fold, p < 0.05). These results indicate that SESN2 responds to both genotoxic and oxidative stress but with cell line-specific sensitivity thresholds.

In contrast, SESN3 exhibited a bifurcated regulatory profile depending on the type of stressor (Figure 9B). DOX induced strong upregulation of SESN3 in both lines, reaching 4.2-fold in KLE (p < 0.001) and 5.22-fold in AN3CA at 4 µM (p < 0.0001). However, menadione treatment led to consistent downregulation of SESN3 in both lines, with expression levels reduced by 20%–50% compared to control across all concentrations tested (p < 0.05), suggesting divergent upstream regulatory mechanisms for oxidative versus genotoxic stress.

SOD1 regulation appeared more variable and context-dependent (Figure 9C). In KLE cells, high-dose DOX (4 µM) significantly upregulated SOD1 expression (3.52-fold, p < 0.05), likely reflecting an adaptive antioxidant response. In contrast, AN3CA cells showed only a marginal and non-significant increase at 4 µM DOX (1.12-fold). Notably, no upregulation was observed at 2 µM DOX in either cell line. Under menadione treatment, AN3CA cells exhibited significant downregulation of SOD1 at both 10 μM and 25 µM concentrations (p < 0.05), suggesting that ROS accumulation may suppress or overwhelm the SOD1-mediated defense pathway in this context.

Together, these data reveal complex transcriptional dynamics of stress-related genes under distinct types of cellular stress. SESN2 emerges as a broadly inducible stress marker responsive to both oxidative and genotoxic stimuli. SESN3, by contrast, appears to be more tightly regulated and differentially expressed depending on the type of damage, implicating divergent upstream signaling cascades. SOD1 expression varies markedly between cell lines and conditions, indicating tightly regulated, stimulus-specific transcriptional control. These findings provide insight into cell-specific redox adaptation mechanisms and identify SESN2, SESN3, and SOD1 as potential markers or therapeutic targets in EC.

## Discussion

In this study, we compared the responses of two EC cell lines, AN3CA and KLE, to DOX and menadione, with a focus on cell viability, ROS generation, and regulation of antioxidant proteins. By employing complementary experimental approaches, ATP-based viability assays, ROS detection, ICW analysis, and qPCR, we identified distinct susceptibility patterns. AN3CA cells were more sensitive to DOX, whereas KLE cells exhibited greater susceptibility to menadione. These cell line–specific differences extended beyond metabolic outcomes and were mirrored in the differential regulation of antioxidant-related genes and proteins.

DOX treatment induced a strong, dose-dependent reduction in viability in AN3CA cells, reaching up to 90% inhibition at 4 μM. This was accompanied by substantial ROS accumulation at 2–4 μM, consistent with the known mechanism of DOX-induced oxidative stress and DNA damage leading to apoptosis (Sarvazyan, 1996; Pilco-Ferreto and Calaf, 2016; Gorini et al., 2018; Montalvo et al., 2020). In contrast, KLE cells showed only moderate viability reduction (∼40%) across the same concentration range, coupled with relatively modest ROS elevation. This phenotype likely reflects greater oxidative tolerance, possibly due to higher basal levels of SOD1 protein in KLE compared with AN3CA. At the protein level, ICW analysis showed that low DOX doses (0.125–0.25 μM) transiently upregulated SESN2, SESN3, and SOD1 in both lines, while higher concentrations led to their suppression. This biphasic response aligns with previous findings demonstrating early adaptive activation of antioxidant defenses, followed by their exhaustion under sustained or excessive oxidative stress (Mai et al., 2016; Xi et al., 2022).

Notably, discrepancies between ICW and qPCR data highlighted the complex regulatory landscape of oxidative stress responses. In AN3CA cells, SESN2 protein levels declined at 2–4 μM DOX, while SESN2 mRNA was upregulated, suggesting post-transcriptional repression. Likewise, SESN3 transcripts were strongly induced in both cell lines under DOX treatment, yet protein levels were suppressed at higher doses. Such transcript–protein mismatches may result from mRNA instability, translational inhibition, or protein turnover delays (Chen et al., 2002). These divergences could also reflect regulatory buffering, such as the long half-life of antioxidant proteins or stabilization mechanisms via Nrf2 signaling. SESN2 is transcriptionally regulated by Nrf2 under oxidative stress, and contributes to redox homeostasis by promoting autophagy and facilitating p62-mediated degradation of Keap1, thereby sustaining Nrf2 activity (Budanov et al., 2010; Lee et al., 2013; Kim et al., 2015). Conversely, SESN3 expression is regulated by the forkhead box O (FOXO) family of transcription factors in response to oxidative stress, with FOXO3-mediated induction implicated in ROS detoxification (Nogueira et al., 2008; Kozak and Jonak, 2023). Additionally, we cannot exclude post-transcriptional regulation by microRNAs (miRNAs). Our previous research demonstrated that SESN2 is targeted by miR-141 and SESN3 by miR-200b/c/429 (Kozak et al., 2019). Several studies have shown that miRNAs play crucial roles in modulating oxidative stress and hypoxic signaling by targeting transcripts for degradation or translational repression (Marsit et al., 2006; Ivan et al., 2008; Mateescu et al., 2011; Snowdon et al., 2011).

Menadione elicited a strong cytotoxic effect in KLE cells (87% viability loss at 25 μM) and in AN3CA cells only at the same high dose (>95% reduction). Interestingly, ROS elevation was significant only in AN3CA cells at 10 μM, whereas in KLE cells, cytotoxicity occurred without detectable ROS increases. This suggests that menadione may act through additional mechanisms, such as glutathione depletion or thioredoxin reductase inhibition, rather than ROS alone (Morin et al., 2008; Li et al., 2019; Sun et al., 2021). ICW data showed stable SESN2, SESN3, and SOD1 protein levels in AN3CA across all doses, while KLE cells displayed significant protein downregulation at 25 μM. At the mRNA level, SESN2 was upregulated in KLE despite reduced protein levels, again indicating post-transcriptional or post-translational regulation. In AN3CA, downregulation of SOD1 and SESN3 transcripts did not translate into decreased protein levels, suggesting maintained protein stability or delayed degradation. These findings reinforce the importance of integrating transcriptomic and proteomic readouts, especially under oxidative stress conditions. SOD1, for example, is a stable protein with a long half-life, and short-term reductions in mRNA expression may not immediately impact its abundance (Trachootham et al., 2008). Moreover, mRNA fluctuations may precede detectable changes in protein levels, particularly within the 24-h window of our experiments (Nguyen et al., 2003; Reichard et al., 2007; Wible et al., 2018).

Together, these results highlight fundamental differences in redox adaptation between AN3CA and KLE cells. KLE cells, with higher basal SOD1 expression, demonstrated stronger antioxidant buffering and reduced sensitivity to DOX-induced ROS. In contrast, AN3CA cells, with lower antioxidant capacity, were more vulnerable to oxidative damage. The distinct regulatory patterns of SESN2 and SESN3 at both transcript and protein levels suggest that multiple, layered mechanisms-including transcriptional, post-transcriptional, and epigenetic—govern redox response. While ICW and qPCR data were not always concordant, their combined use provided a more complete picture of redox signaling in EC cells.

## Limitations

Several limitations should be acknowledged. First, all experiments were performed exclusively in established EC cell lines maintained in 2D culture. While this approach enables controlled mechanistic comparisons, it does not recapitulate tumor heterogeneity or the influence of the tumor microenvironment (e.g., stromal/immune interactions and extracellular matrix), which are known to affect anticancer drug efficacy. Therefore, our findings should be interpreted in the context of these *in vitro* models and validated in more physiologically relevant systems, such as 3D spheroids/organoids and co-culture platforms, and ultimately *in vivo*. Second, ROS measurements were conducted only at a 24-h timepoint, which may not capture earlier ROS peaks, particularly for menadione. Third, ICW normalization using CellTag 520 may be confounded by apoptosis-induced morphological changes under high cytotoxic stress, potentially leading to underestimation of protein levels. Fourth, the absence of ROS modulators (e.g., H_2_O_2_, NAC) limits mechanistic interpretation. Fifth, differential sensitivity may also be influenced by cell line-specific differences in drug uptake and efflux. Variable activity of ABC transporters (e.g., ABCB1/P-glycoprotein) can modulate intracellular DOX accumulation and thereby affect apparent sensitivity; similar considerations may apply to menadione. This was not directly assessed here and should be addressed in future studies by quantifying intracellular drug levels and/or using transporter inhibitors. Finally, while mRNA–protein discrepancies suggest post-transcriptional regulation, these mechanisms were not directly investigated and warrant further exploration.

## Strengths

The study’s primary strength lies in its integrative design, combining functional and molecular assays- ATPlite, CellROX, ICW, and qPCR-to assess cell-specific redox responses. The use of two clinically relevant EC cell lines (AN3CA and KLE), both TP53-mutated and hormone-independent, enhances translational significance. Application of the ICW method offered high reproducibility and throughput, enabling precise quantification of protein dynamics *in situ*. Antibody optimization further increased the robustness of ICW data. Additionally, the inclusion of multiple doses and replicates allowed reliable detection of dose-dependent and biphasic stress responses.

## Conclusion

This study reveals distinct redox adaptation strategies in AN3CA and KLE endometrial cancer cells under chemotherapeutic and oxidative stress. AN3CA cells are more susceptible to DOX-induced ROS accumulation, whereas KLE cells demonstrate stronger basal antioxidant defenses but are vulnerable to high-dose menadione. Discrepancies between mRNA and protein expression of SESN2, SESN3, and SOD1 point to the critical role of post-transcriptional regulation. These findings underscore the importance of integrating multi-level molecular data to capture the complexity of oxidative stress adaptation and highlight SESN2, SESN3, and SOD1 as promising targets for modulating redox balance in EC.

## Data Availability

The raw data supporting the conclusions of this article will be made available by the authors, without undue reservation.
